# Exploring the Pharmacological Mechanisms of Xihuang Pills Against Prostate Cancer *via* Integrating Network Pharmacology and Experimental Validation *In Vitro* and *In Vivo*

**DOI:** 10.3389/fphar.2021.791269

**Published:** 2022-03-07

**Authors:** Yongrong Wu, Xujun You, Qunfang Lin, Wei Xiong, Yinmei Guo, Zhen Huang, Xinjun Dai, Zhengjia Chen, Si Mei, Yan Long, Xuefei Tian, Qing Zhou

**Affiliations:** ^1^ College of Integrated Traditional Chinese and Western Medicine, Hunan University of Chinese Medicine, Changsha, China; ^2^ Graduate School of Hunan University of Chinese Medicine, Changsha, China; ^3^ Shenzhen Baoan District Hospital of Traditional Chinese Medicine, Shenzhen, China; ^4^ Surgery of Traditional Chinese Medicine, The First Affiliated Hospital of Hunan University of Chinese Medicine, Changsha, China; ^5^ Hunan Provincial Key Laboratory of Traditional Chinese Medicine Prescription and Transformation, Hunan University of Chinese Medicine, Changsha, China; ^6^ Department of Physiology, Faculty of Medicine, Hunan University of Chinese Medicine, Changsha, China; ^7^ Hunan Provincial Key Laboratory of Chinese Medicine Oncology, Changsha, China

**Keywords:** prostate cancer, Xihuang pills, integrated pharmacological, mechanism, Chinese medicine, PI3K/AKT/mTOR

## Abstract

**Background:** Drug resistance is the major cause of increasing mortality in prostate cancer (PCa). Therefore, it an urgent to develop more effective therapeutic agents for PCa treatment. Xihuang pills (XHP) have been recorded as the efficient anti-tumor formula in ancient Chinese medical literature, which has been utilized in several types of cancers nowadays. However, the effect protective role of XHP on the PCa and its underlying mechanisms are still unclear.

**Methods:** The active ingredients of XHP were obtained from the Traditional Chinese Medicine Systems Pharmacology Database and Analysis Platform (TCMSP) and BATMAN-TCM. The potential targets of PCa were acquired from the Gene Cards and OMIM databases. R language and Perl language program were utilized to clarify the interaction between the PCa-related targets and the potential targets of XHP. The potential targets of XHP for prostate cancer were gathered from the Gene ontology and KEGG pathway. Furthermore, cell proliferation assays were verified by PC3 and LNCaP cells. The efficacy and potential mechanism tests were confirmed by the PCa PC3 cells and mice subcutaneous transplantation. The effects of PI3K/Akt/mTOR-related proteins on proliferation, apoptosis, and cell cycle of PCa cells were measured by the Cell Counting Kit-8(CCK8), TUNEL assay, real-time quantitative reverse transcription PCR (QRT-PCR), and Western Blotting, respectively.

**Results:** The active components of four traditional Chinese medicines in XHP were searched on the TCMSP and Batman TCM database. The biological active components of XHP were obtained as OB ≥30% and DL ≥0.18. The analysis of gene ontology and KEGG pathway identified the PI3K/Akt/mTOR signaling pathway as the XHP-associated pathway. Collectively, the results of *in vitro* and *in vivo* experiments showed that XHP had the effect of inhibiting on the proliferation of PC3 and LNCaP cells. XHP promoted the apoptosis and restrained the cell cycle and invasion of the PC3 cells and subcutaneous transplantation. Meanwhile, the suppression of XHP on the level of expression of PI3K, Akt, and mTOR-pathway-related pathway proteins has been identified in a dose-dependent manner.

**Conclusion:** PI3K/Akt/mTOR pathway-related pathway proteins were confirmed as the potential XHP-associated targets for PCa. XHP can suppress the proliferation of prostate cancer via inhibitions of the PI3K/Akt/mTOR pathway.

## Introduction

Prostate cancer is a life-threatening malignant tumor, ranking first in morbidity and second in mortality among males (Siddall et al., 2020; Van den Broeck et al., 2020). Prostate cancer has obscure symptoms and usually progresses to the advanced stage while obvious symptoms emerge (Layman et al., 2019). Advanced prostate cancer, especially castration-resistant prostate cancer (CRPC), is characterized by high metastasis and aggressiveness and drug resistance, leading to the terminal stage of prostate cancer (Armstrong and Gao, 2015; Hadaschik and Hellmis, 2021). Therefore, it is an urgent demand for new effective treatment of prostate cancer.

The excavation of antitumor drugs from Traditional Chinese medicine (TCM) has been high-profile in recent years (Wang et al., 2020). Xihuang pills (XHP) are an anti-tumor Chinese patent medicine applied in China, which is composed of musk, bezoar, frankincense, and myrrh. It was first recorded in the ancient books such as the *Wai Ke Quan Sheng Ji* compiled by Wang Weide in the Qing Dynasty. Furthermore, it is recorded that it can be applied in malignant diseases such as Shi-ju, Ru-yan, and E-he. Nowaday, XHP have been used in the treatment of various cancers, such as breast cancer, lung cancer, lymphoma, and so on, and have achieved certain efficacy (Fu et al., 2020; Li et al., 2020; Xu X et al., 2020). The antitumor mechanism of XHP is *via* inducing tumor cell apoptosis (Su et al., 2018), inhibiting tumor cell proliferation (Hao et al., 2018), blocking the cell cycle, and regulating tumor microenvironment (Yang et al., 2020), etc. Especially for the treatment of breast cancer, XHP combined with endocrine and chemotherapy drugs can improve the quality of life and survival time of breast cancer patients (Taitt, 2018). Prostate cancer is an androgen-related malignant tumor (Zhang et al., 2018), and endocrine therapy is also an important treatment for advanced prostate cancer (Stoykova and Schlaepfer, 2019). Therefore, it will be of significance to explore the effect and related mechanism of XHP on prostate cancer.

TCM has the pharmacological characteristics of multiple targets (Hui et al., 2020). In order to seek the potential targets of XHP, we applied the network pharmacology method to analyze network biocommunication prediction and the targets of XHP to treat prostate cancer. First of all, network pharmacology technologies were utilized to search the effective active components of XHP and the targets of XHP to treat prostate cancer. Furthermore, the gene ontology and KEGG pathway enrichment analysis were applied to predict the targets of XHP to treat prostate cancer. Finally, the cell and animal experiments were wielded to verify the potential targets predicted by network pharmacology methods. The PI3K/Akt signaling pathway is one of the apoptosis signaling pathways of vascular endothelial cells, and the abnormal activation of this pathway is the basis of the formation of abnormal vascular network (Cottrell et al., 2021). Several clinical and experimental studies have shown that XHP can inhibit angiogenesis in tumor cells and tissues such as breast cancer and gastric cancer (Wu et al., 2020; Wang et al., 2021). Therefore, this paper researched the effect of XHP on the changes of PI3K/Akt/mTOR signaling pathway in prostate cancer; meanwhile, the immunohistochemistry combined with immunofluorescence technology was used to verify the effect of XHP on the angiogenesis of prostate cancer and the level of angiogenic factors. This study will provide experimental basis for explaining the mechanism of XHP in the intervention of prostate cancer.

## Methods

### Network pharmacology-based analysis

#### Screening of active components and construction of the interaction network in XHP

The active components and target genes of XHP were identified by the TCMSP database (https://old.tcmsp-e.com/tcmsp.php) and BATMAN-TCM database (https://bionet.ncpsb.org.cn/batman-tcm). Then, the bioactive components of XHP were determined as those screened with oral bioavailability (OB) ≥30% and drug likeness (DL) ≥0.18 as the limiting conditions. The active component targets were constructed by Cytoscape 3.7.1 Software (https://cytoscape.org/).

#### Screening of disease targets for prostate cancer

The Genecards database (https://www.genecards.org/) and OMIM database (https://www.omim) were used for searching the targets of “prostate cancer.”

#### Venny diagram and PPI network construction

Venny (https://bioinfogp.cnb.csic.es/tools/venny/index.html) was used to clarify the interaction between prostate cancer-related targets and potential targets of XHP. A common target interaction network (i.e., PPI network) was built by the STRING (https://cn.string-db.org/) plug-in (protein–protein interaction, PPI) with the protein type of “*Homo sapiens*,” and the setting was “high confidence: 0.7.” The count R plug-in was applied to obtain the frequency of common protein targets.

#### Construction of the interaction network of the XHP-based active ingredient for the prostate cancer

The interaction network for the active ingredient of XHP and prostate cancer was set up by the Cytoscape 3.7.1 Software (https://cytoscape.org/). In the network diagram, “node” represents the active ingredient and the target, and “edge” represents the relationship between the active ingredients of XHP and the prostate cancer-associated target.

#### Gene ontology (GO) analysis and KEGG pathway enrichment

GO analysis is mainly used to describe the function of gene products, including the cellular function, molecular function, and biological function. *R* language (https://www.r-project.org/) was utilized to analyze the common targets of XHP-based active ingredients and prostate cancer. The cluster profiler KEGG was used for KEGG path enrichment analysis. According to the enrichment factor value, the enrichment degree of the core pathway was identified, which helps to confirm the possible XHP-based anti-tumor signaling pathways of prostate cancer.

#### Ultraperformance liquid chromatography coupled with quadruple time-of-flight mass spectrometry assay

The qualitative analysis of components in XHP was employed by the LC-MS/MS method. Twenty pieces of XHP were fetched, ground into a powder with an accurate measurement of 2.00 g of XHP power, dissolved in 20 ml 100% methanol (HPLC grade), filtered through a 0.22 µm membrane filter, centrifuged at 8,000×*g*, for 5 min, and finally collected the supernatant for analysis. Analysis was carried out on a common C18 column (Agilent ZORBAX Eclipse Plus C18, 3.0 × 100 mm, 1.8 μm) and the column temperature was maintained at 25°C. The mobile phase was composed of A (0.1% formic acid in water, v/v) and B (acetonitrile) using a gradient elution of 5%–15% B at 0–5 min, 15%–25% B at 5–10 min, 25%–45% B at 10–20 min, 45%–65% B at 20–30 min, and 65%–95% B at 30%–40 min. The flow rate was set at 0.4 ml/min. The injection volume was 2 μl. The accurate mass spectrometric experiment was operated in the ESI negative and positive-ion mode of the Agilent 1290UPLC-6540-Q-TOF accurate mass spectrometer system equipped with an ESI ion source (Agilent Technologies, Santa Clara, CA, USA). The following operation parameters were used: ion source gas 1 and ion source gas, 210 psi; curtain gas, 35 psi; ion spray voltage floating, 4000 V; temperature, 350°C; collision energy, 110 V; and collision energy spread, 30 V. Finally, the data was managed with MFE (molecular feature extract) of Agilent Masshunter Qualitative Analysis (Agilent). Components in XHP were referred to the mass spectrometric data of the standard substance by consulting the reference literature.

## Experimental verification

### Cell lines and reagents

The human prostate cancer cell lines PC3 (cell identification number: 20160628-01) and LNCaP (cell identification number: 20180528-01) were obtained from Beijing Beina Chuanglian Biotechnology Institute; XHP (approval number: z11020073, batch number: 17043278), Beijing Tong Ren Tang, Beijing, China; DMEM F12 medium, GIBCO, Grand Island, NY, USA; fetal bovine serum (FBS), GIBCO; CCK-8, GIBCO; 3-MA (IM0190), Beijing Solarbio Technology Co., Ltd.; VEGFR (Ab 133273), Abcam, Cambridge, UK; αSMA (Ab7817) Abcam; PI3K (60225-1-ig), Proteintech, Chicago, IL, USA; Akt (Ab185633), Abcam; mTOR (Ab32028), Abcam; p-PI3K (ab182651), Abcam; p-Akt (ab192623), Abcam; p-mTOR (Ab137133), Abcam; cleaved caspase3 (ab214430), Abcam; caspase9 (ab202068), Abcam; PCNA (10205-2-AP), Proteintech; and β-actin (66009-1-Ig), Proteintech.

### Preparation of XHP extract and inhibitor solution for cell experiment

XHP 3 g was dissolved in 6 ml double distilled water for 24 h. The mixture was dissolved with the ultrasonic dissolver for 2 min. Then, the solubilized suspension was centrifuged at 6,000 rpm for 10 min. Both the supernatant and sediment were collected. The collected sediment was further dissolved in 6 ml DMSO with an ultrasound device, followed by 6,000 rpm centrifugation for 10 min. The XHP solution with the final concentration of 500 mg/ml was prepared by mixing the supernatant from the first and second centrifugation. The prepared solution was stored at 4°C for future use. In the *in vitro* assay, the solution was diluted to 5, 2.5, 1.25, and 0.625 mg/ml (crude dose) with the serum-free medium, respectively. According to the instructions, 5 mg 3-MA was dissolved in 3.2523 ml DMSO to the final concentration of 10 mM. [Woo Y, Jung YJ. Angiotensin II receptor blockers induce autophagy in prostate cancer cells. Oncol Lett. 2017 May; 13(5):3579-3585].

### Cell viability assay

PC3 and LNCaP cells (5,000 cells/well) were seeded in 96-well plates and treated with different concentrations of XHP (0.625, 1.25, 2.5, and 5 mg/ml) for 6, 12, 24, or 48 h, then 10 μl of Cell Counting Kit-8 (CCK-8) solution was added to each well and the cells were cultured at 37°C for 2 h. The cell viability was confirmed by reading the absorbance at 450 nm in a microplate reader (TECAN, Switzerland).

### Flow cytometry for cell cycle analysis

PC3 cells were seeded in the T25 culture flask and incubated with or without XHP (1.25 mg/ml) at 37°C. After incubation, cells were collected and fixed with 70% ethanol at 4°C for overnight, followed by staining with 50 μg/ml propidium iodide (Sigma, St. Louis, MO, USA) for 45 min in the dark. A Canto II flow cytometer (Beckman, A00-1-1102) was utilized for cell cycle analysis.

### Flow cytometry for cell apoptosis

PC3 cells were seeded in the T25 culture flask and incubated with or without XHP (1.25 mg/ml) at 37°C for 24 h. Then, cells were collected and fixed with precooled 4% paraformaldehyde. After washing with PBS, the fixed cells were digested with 0.25% trypsin (excluding EDTA), and centrifuged at 2000 rpm for 5 min. After a further PBS washing step, the collected cells were mixed with 500 μl binding buffer. Then, 5 μl propidium iodide and Annexin V-APC (Nanjing Kgi, KGA1030-1007) were added and the mixture was placed in the dark at room temperature for 5–15 min. A Canto II flow cytometer (Beckman, A00-1-1102) was utilized for apoptosis analysis.

### Tumor cell invasion assay

Serum-free medium 300 μl was mixed with 50 μl matrix gel. 5 × 10^5^ PC3 cell suspension 100 μl was placed into the upper chamber of the Transwell chamber; meanwhile, 500 μl fetal bovine serum was added into the lower chamber of the Transwell chamber. After being cultured for 24 h, the chamber was fixed with 4% paraformaldehyde at 4 C. Finally, 0.1% crystal violet (G1061, Solarbio) was added and incubated for 30 min at room temperature. After being cleaned with PBS twice, the cells on the upper surface of the chamber were gently wiped with cotton balls and observed and counted under a microscope (ZEISS, Oberkochen, Germany). The experiment was repeated thrice.

### Preparation of XHP suspension for animal experiment

The daily dosage of XHP for the adults is 6 g/d, which equals to 78 mg/kg for the mouse. The calculation is based on the ratio of body surface area between human and mouse. A 5% DMSO XHP extract suspension was prepared for animal study.

### Prostate cancer subcutaneous transplantation mice model

A subcutaneous transplantation mice model was established to further verify the anti-proliferative effect of XHP on prostate cancer. The study protocols were in accordance with the European Community guidelines on the use and care of laboratory animals and were approved by the Ethics Committee of the Central South University. The mice model of subcutaneous transplantation was established by PC cells injection. In general, 1×10^7^ PC3 cells were subcutaneously injected into the middle and rear armpit of the BALB/C male nude mice. As the average volume of subcutaneous transplantation reached to 200 mm^3^, the mice were randomized into the two groups, including the controls and the XHP treatment group (n = 6). The volume was calculated as (length × width × height) × π/6 (Kim and Kim, 2008). In the test group, mice received the XHP for 78 mg powder/kg per day for 2 weeks, whereas controls received the equal volume of normal saline. After treatment, tumor tissues were collected from the sacrificed mice. The tumor body weight inhibition rate (%) was calculated as [(average tumor volume in control group − average tumor volume in administration group)/average tumor volume in control group] × 100%. (Liu et al., 2015).

### Histopathological examination

Tumor tissues were dissected into slides, fixed with 4% formaldehyde, and observed under an optical microscope. Paraffin slices were prepared as well, and the VEGFR and αSMA protein expression in the subcutaneous transplantation of prostate cancer tissues and the corresponding tissues were assessed with a Immunofluorescence 3′-diaminobenzidine (DAB) kit using the anti-VEGFR (Ab 133273; Abcam) and anti-αSMA (Ab7817; Abcam) monoclonal antibodies, respectively.

### Western blotting

The protein concentration of the PC3 cells and tumor tissues was confirmed by the BCA protein assay (ComWin Biotech, Beijing, China). The proteins were separated by 12% SDS-PAGE and transferred to the polyvinylidene fluoride (PVDF) membrane. After blocking with the 5% skim milk, the membranes were incubated with the primary antibodies (anti-PI3K, anti-Akt, anti-mTOR, anti-p-PI3K, anti-p-PI3K, anti-p-Akt, anti-p-mTOR, anti-cleave-Caspase-3, anti-Caspase-9, and anti-PCAN antibody at 1:1000) at 4°C overnight. After washing with TBST, the membranes were incubated with the secondary antibodies (HRP conjugated-goat anti-mouse IgG, SA00001-1; Proteintech) for 2 h at room temperature. Immunoreactivity was determined by an advanced ECL kit (AWB0005b; Abiowell) and visualized using a chemiluminescence imaging system (Chemiscope 6,100; Clinx Science Instruments).

### Real-time quantitative polymerase chain reaction (qRT-PCR)

Total RNA was extracted with TRIzol^®^Reagent (TIANGEN BIOTECH, Beijing, China) and reverse-transcribed with NovoScript^®^ Plus all-in-one first-strand cDNA Synthesis SuperMix (gDNA Purge) Reverse Transcriptase (Novoprotein, Shanghai, China) according to the manufacturer instructions. The primers were synthesized by the Sangon Biotech (Shanghai), and were listed in Table 1.

**TABLE 1:** Primer design

Gene	Primer sequence	Product length (bp)	Website
M-actin	F:ACATCCGTAAAGACCTCTATGCC	223	http://www.ncbi.nlm.nih.gov/gene/11461
	R:TACTCCTGCTTGCTGATCCAC		
M-PI3K	F:CGAGAGTGTCGTCACAGTGTC	122	https://www.ncbi.nlm.nih.gov/nucleotide/1720372125
	R:TGTTCGCTTCCACAAACACAG		
M-Akt	F:CCCTGCTCCTAGTCCACCA	85	https://www.ncbi.nlm.nih.gov/nucleotide/1720423932
	R:TGTCTCTGTTTCAGTGGGCTC		
M-MTOR	F:CCGCTACTGTGTCTTGGCAT	118	https://www.ncbi.nlm.nih.gov/gene/56717
	R:CAGCTCGCGGATCTCAAAGA		
M-casp3	F:TCTGACTGGAAAGCCGAAACTCT	100	https://www.ncbi.nlm.nih.gov/gene/12367
	R:AGCCATCTCCTCATCAGTCCCA		
M-casp9	F CAC​CTT​CCC​AGG​TTG​CCA​AT	153	https://www.ncbi.nlm.nih.gov/gene/12371
	R GCC​ATG​AGA​GCT​TCG​GAG​AG		

### Statistical analysis

SPSS 22.0 (IBM, Armonk, NY, USA) was used for statistical analysis, and the data was expressed as $\bar{x}\pm s$ . The GraphPad Prism 7 (GraphPad Software, San Diego, CA, USA) was employed for plotting. *t*-Test was utilized to compare the difference between two groups of the independent sample which conformed to normal distribution. Kruskal–Wallis test was employed to compare the difference between two groups of the independent sample which was inconsistent with normal distribution. One-way ANOVA was used to compare the difference among multiple groups. The LSD test was utilized for pairwise comparison among multiple groups. All tests were two-sided tests, and p ≤ 0.05 or p ≤ 0.01 was considered statistically significant.

## Results

### Construction of active ingredient target interaction network

**FIGURE 1:**
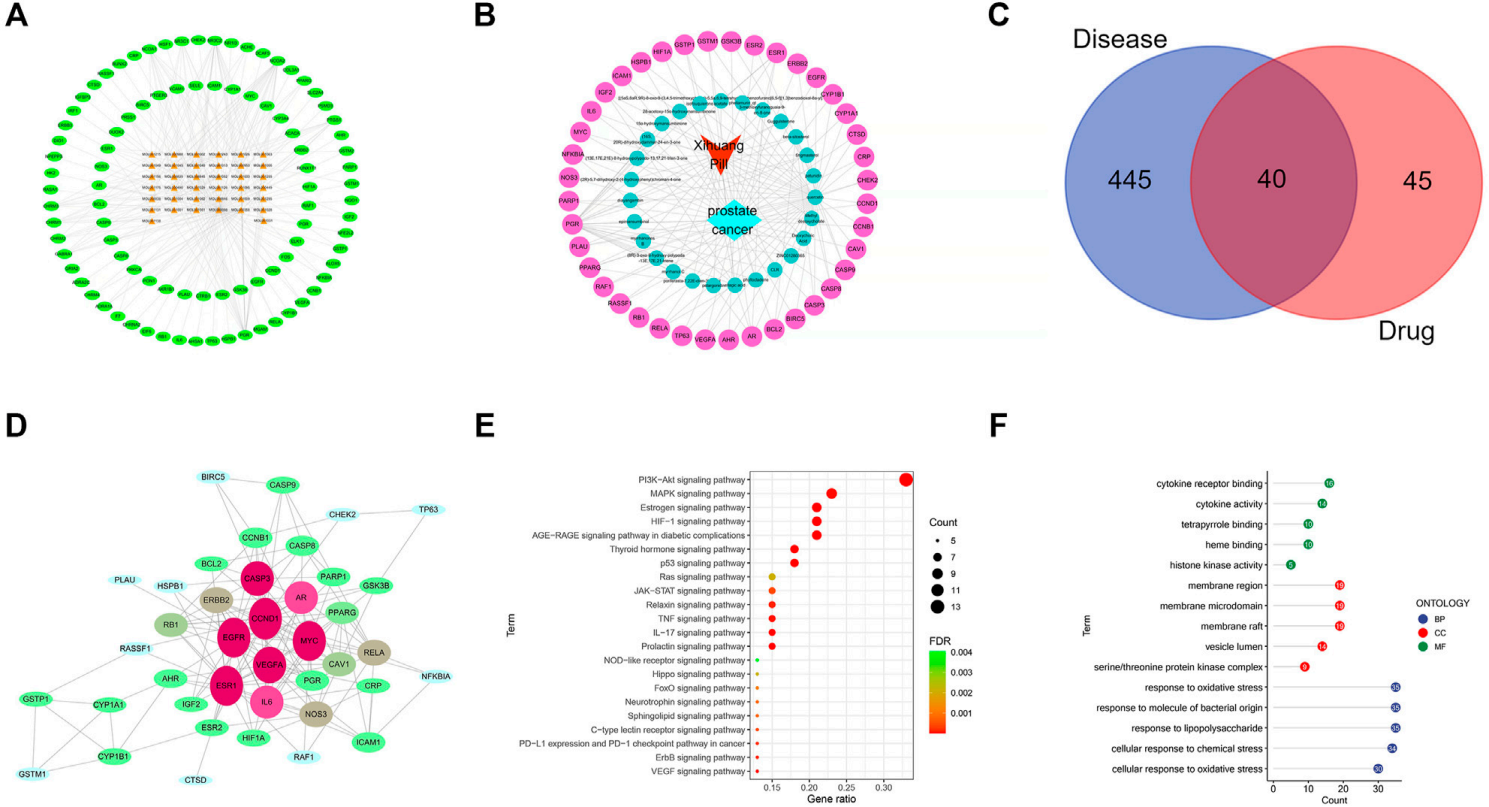
The process of this research (IF, Immunofluorescence).

The bioactive components of XHP were determined as those screened with oral bioavailability (OB) ≥30% and drug likeness (DL) ≥0.18 as the limiting conditions on the TCMSP and BATMAN-TCM databases. Twenty-seven bioactive components (ellagic acid, pelargonidin, quercetin, etc.) of XHP were obtained by the software of Cytoscape 3.7.1, and 206 targets (without duplication) of the active components were acquired to construct the active component target interaction network. The bioactive ingredients were represented by the orange nodes and the potential targets of bioactive ingredients were represented by the green nodes. Degree indicated the number of routes connecting nodes in the network. The active ingredients with high degree value play an important role in the pharmacological function of XHP. The top three MOL 000098 (quercetin) degree was 77; MOL000358 [β-sitosterol, the degree of sitosterol was 16; MOL 000449 (stigmasterol) degree was 13] (Figure 1A).

### Construction of active ingredient prostate cancer target interaction network

Sixty-nine nodes (40 targets, 27 active ingredients, 1 disease, and 1 drug) and 147 edges were obtained in the active ingredient prostate cancer target interaction network diagram by the Cytoscape 3.7.1 software. The red, light blue, green, and light red nodes represented the XHP, prostate cancer, active ingredients of XHP, and the common target of active ingredients of XHP and prostate cancer, respectively (Figure 1B).

### Common target screening and interaction network construction

Four hundred eighty-five therapeutic targets of prostate cancer were obtained by setting the correlation score ≥17.0 and deleting the repeated targets on gene cards and OMIM database; 485 targets for prostate cancer and 85 targets (including repetition) for drug active ingredients were screened out by R language software and Perl language program. A total of 40 targets were input into the Venny 2.1 software. The Venny diagram obtained was shown in Figure 1C. The string data platform was employed to input 40 common targets, and the PPI was constructed in the mode of “high confidence: 0.7” (Figure 1D). The top 30 protein targets with high frequency were CCND1, EGFR, ESR1, and MYC, which can be used as the potential targets of XHP in the treatment of prostate cancer.

### GO function analysis and core pathway screening of XHP in the treatment of prostate cancer

**FIGURE 2:**
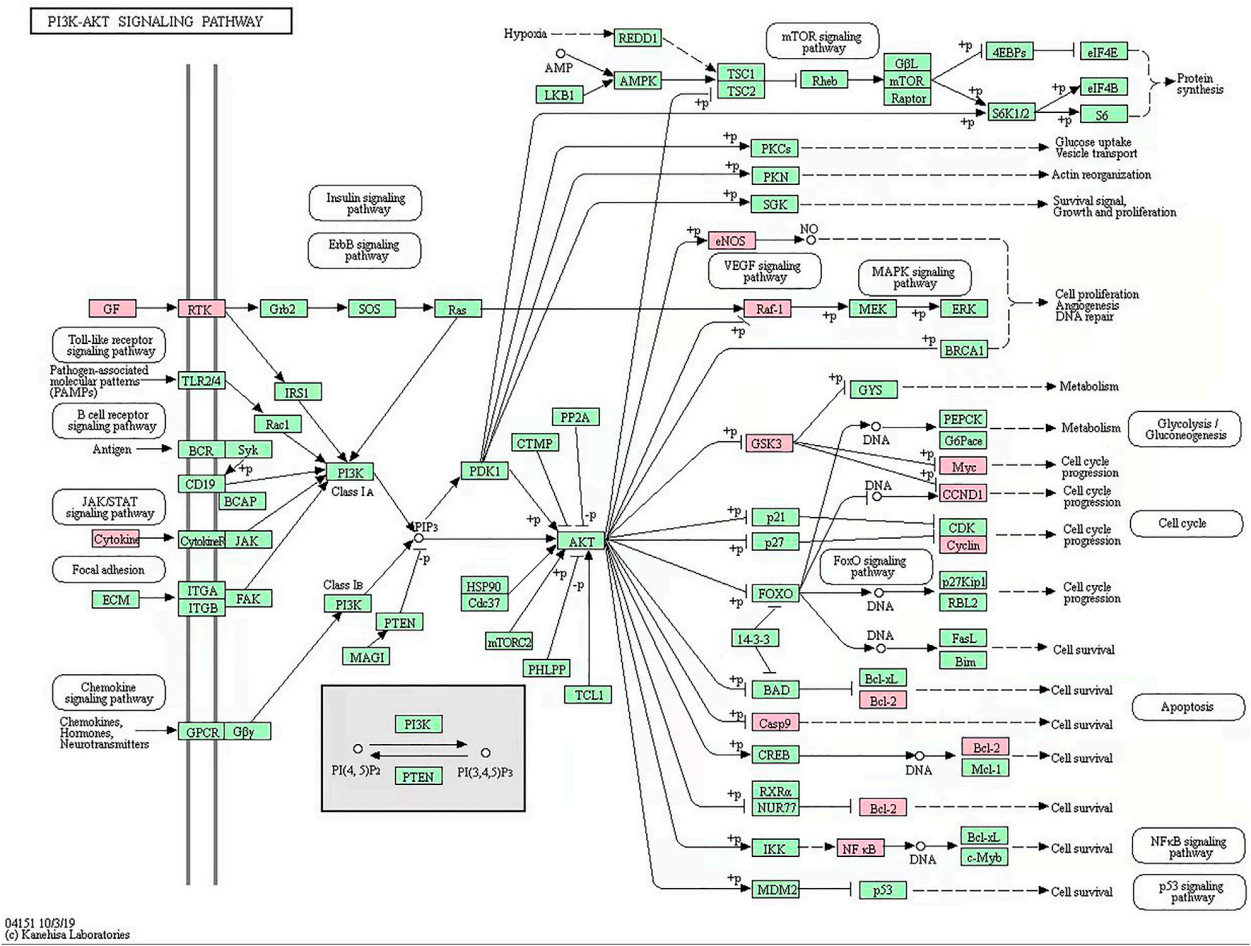
Xihuang pills acts on potential target of PI3K-Akt signaling pathway. The green node represents a potential target for the action of Xihuang pills or an enzyme and compound associated with a potential target.

R language software was used to analyze the go function of the above common targets. The bubble diagram and lollipop diagram are shown in Figures 1E,F, respectively. The biological processes were mainly related to ubiquitination of protein bodies, heterodimerization activity of proteins, and activity of transcription factors. XHP may regulate multiple complex biological processes to treat prostate cancer. The signal pathways with more than five enriched targets are listed in Table 2. Forty common targets are mainly distributed in PI3K-Akt, MAPK, AGE-RAGE, HIF-1, estrogens, and other signaling pathways, which suggests that XHP can treat prostate cancer by acting on multiple signaling pathways. The potential target of XHP in the treatment of prostate cancer based on the PI3K-Akt signaling pathway is shown in Figure 2.

**TABLE 2:** Signal pathway with KEGG target number ≥5 of Xihuang pills

ID	Signal pathway	Number of genes	Genes
hsa04151	PI3K-Akt signaling pathway	13	RELA/VEGFA/IGF2/BCL2/CASP9/GSK3B/EGFR/CCND1/IL6/RAF1/ERBB2/MYC/NOS3
hsa04010	MAPK signaling pathway	9	RELA/VEGFA/IGF2/CASP3/EGFR/RAF1/ERBB2/MYC/HSPB1
hsa04933	AGE-RAGE signaling pathway in diabetic complications	8	RELA/VEGFA/BCL2/CASP3/CCND1/IL6/ICAM1/NOS3
hsa04066	HIF-1 signaling pathway	8	RELA/VEGFA/BCL2/EGFR/IL6/HIF1A/ERBB2/NOS3
hsa04915	Estrogen signaling pathway	8	ESR1/PGR/BCL2/ESR2/EGFR/RAF1/NOS3/CTSD

**FIGURE 3:**
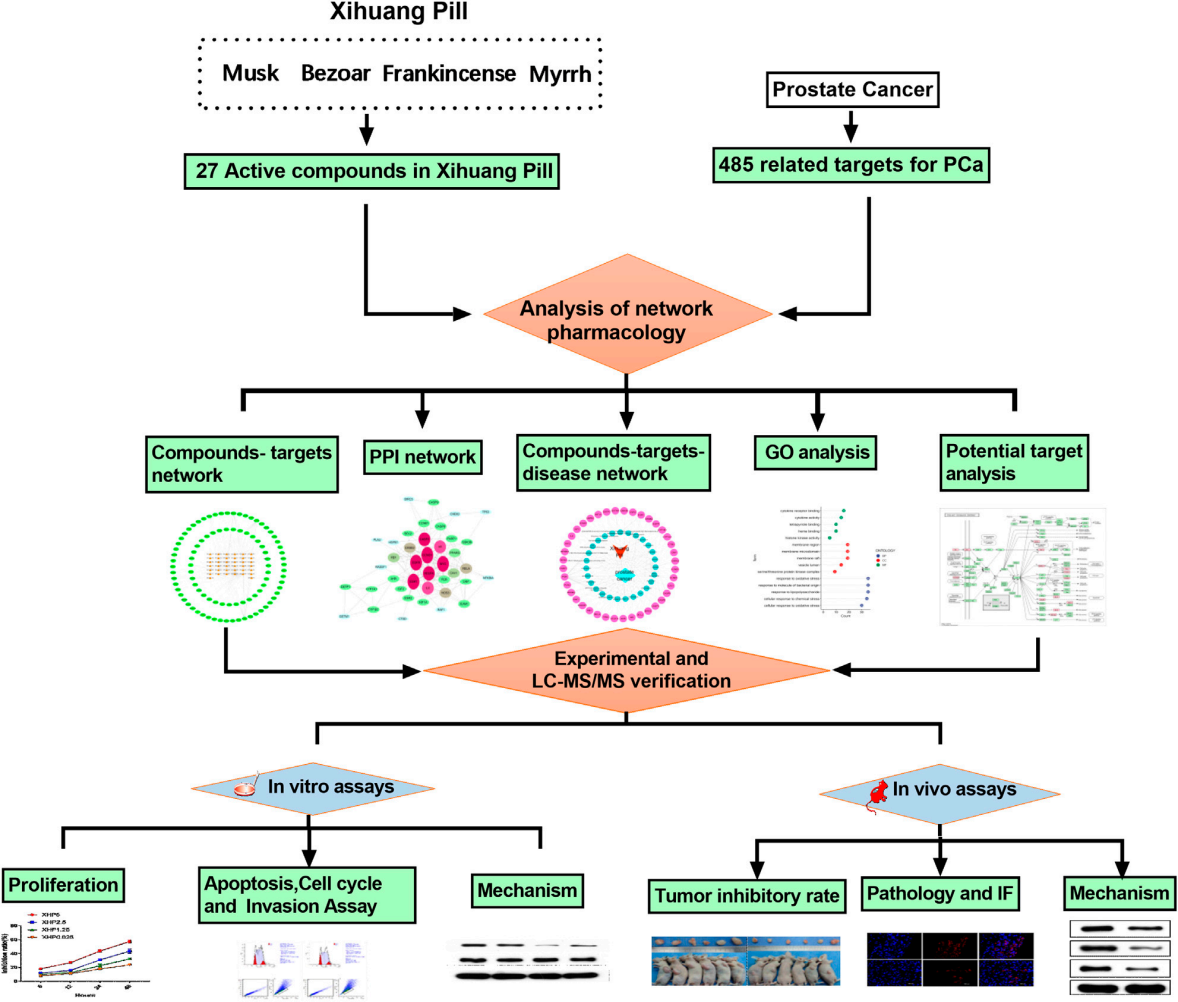
The process of this research. This study was divided into three main sections:1) Prediction of the targets of XHP for prostate cancer by network pharmacology; 2) Ultraperformance liquid chromatography coupled with quadruple time-of-ight mass spectrometry assay of XHP; 3) Experimental validation in vitro and in vivo to verify the mechanism of XHP in the treatment of prostate cancer was via PI3K/Akt/mTOR pathway.

According to the results of the network pharmacology, the potential mechanism of XHP to treat prostate cancer was via PI3K/Akt/mTOR signaling pathway. Next, the ultraperformance liquid chromatography coupled with quadruple time-of-ight mass spectrometry assay was used to control the quality of XHP. Finally, the experiments in vitro and in vivo were employed to verify the mechanism of XHP to treat prostate cancer (Figure 3).

### Total ion flow diagram of XHP by LC-MS/MS

**FIGURE 4:**
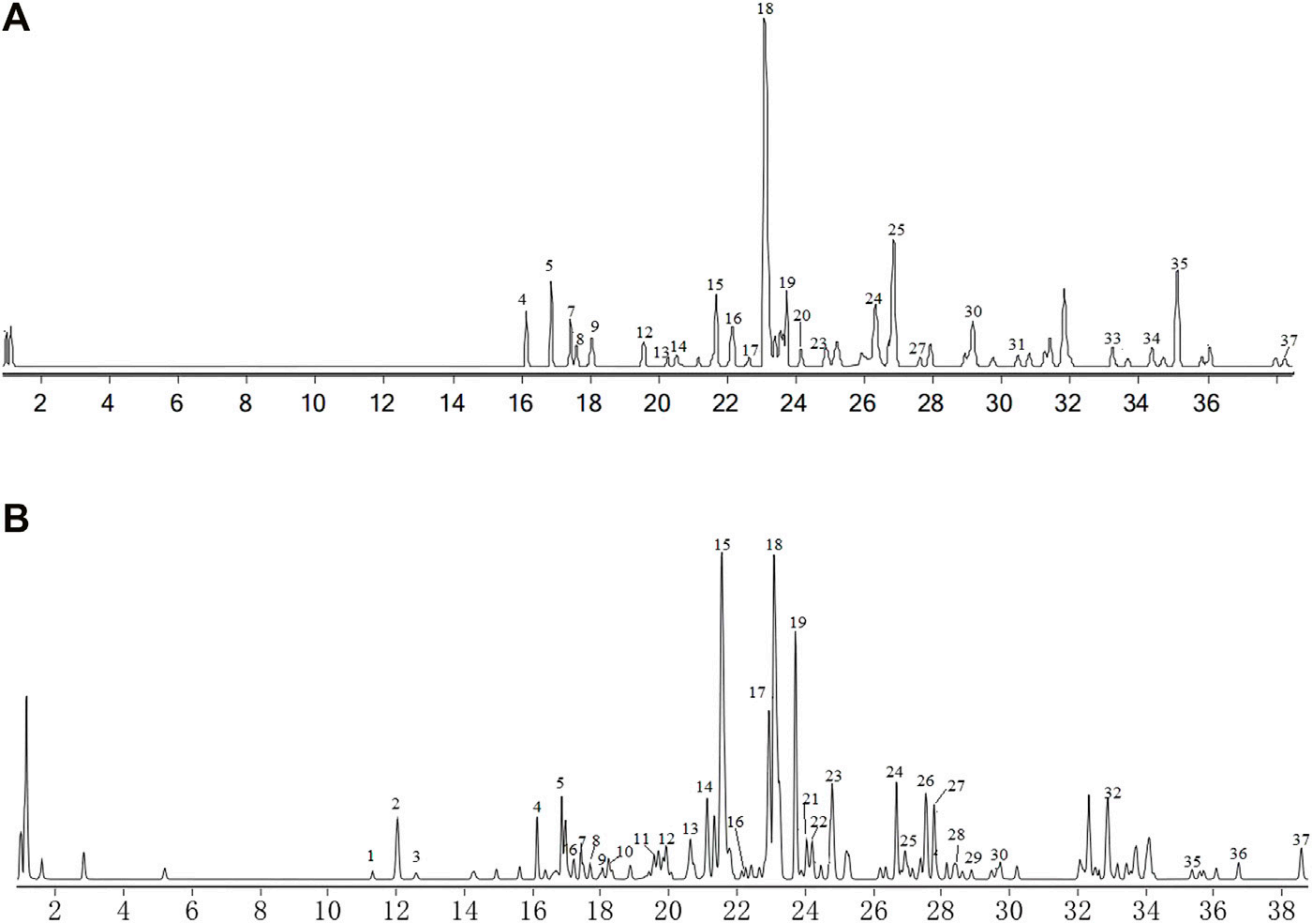
Total ion chromatograms (TIC) from XHP. **(A)** TIC (+ESI TCC) of XHP. **(B)** TIC (-ESI TCC) of XHP. Figure 1 Total ion chromatograms (TIC) from XHP. **(A)** TIC (+ESI TCC) of XHP. **(B)** TIC (-ESI TCC) of XHP.

Qualitative analysis of components XHP was performed by the LC-MS/MS method. Chromatograms of total ion in ESI positive and negative-ion mode for XHP are shown in Figures 4A,B. We preliminarily identified 37 compounds in this extract. They were D-9-AnthrylaAlanine,Trimethylolpropane triacrylate, Subaphyllin, 1,3,4-Oxadiazol-2(3H)-one, Octylacetate, Curzerenone, Ethyl2-acetamido-2-deoxyhexopyranoside, rel-1S, 2S-epoxy-3R-methoxy-4R-furanogermacr-10(15)-en-6-one, palmitic acid, 1,3,4-Oxadiazol-2(3H)-one, Taurocholic acid, Ricinoleic acid, Abietic acid, Ethylenediamine Diaceturate, Rhodomollein III, Myrrhone, glycocholic acid, Decyl acetate, Lauric Acid Isobutyl Ester, Myrrhone, 9,10-Dihydroxystearic acid, taurodeoxycholic acid, Cholic acid, Glycochenodeoxycholic acid, 1-Octanol, Oleic acid, Abietic acid, Fritillebic acid, Broussonin B, [2-(2-phenylethyl)phenyl]methanol, Eicosanetetraenoic acid, curzerenone, Myrrhone, 11-Oxo-β-boswellic acid, Diacetoxytetrahydroxytaxadiene, Anacardic acid B, and Acetyl-11-keto-β-boswellic acid, as shown in Table 3.

**TABLE 3:** List of main components of XHP mass spectrometry

Nub	tR/min	PIM	AM	Error	MGF	Mass	Name
1	12.307		M + CH3COOH = 325.1322	−0.8	C17H15NO2	256.1103	D-9-AnthrylaAlanine
2	12.584		M − H = 295.1214	0.29	C18H18NO3	296.1297	Trimethylolpropane triacrylate
_3_	14 952		M + COOH = 309 1448	1 28	_C14H20N2O3_	264 1474	Subaphyllin
4	17.343	M + H = 263.1257		1.32	C15H18O4	262.1184	1,3,4-Oxadiazol-2 (3H)-one
5	17 69	M + Na = 195 1364		−1 89	C10H20O2	172 1463	Octyl acetate
6	18 038	M + H = 231 1371		3 92	C15H18O2	230 1271	Curzerenone
7	18.895		M + CH3COOH = 309.1417	2.83	C10H19NO6	249.1212	Ethyl 2-acetamido-2-deoxyhexopyranosid e
8	19.204	M + H = 247.1319		4.23	C15H18O3	246.1256	rel-1S,2S-epoxy-3R-methoxy-4R-furanogermacr-10 (15)-en 6 one
9	20 520	M + Na = 279 2,287		3 24	C16H32O2	256 2,402	Palmitic acid
10	21.330		M − H = 261.1161	−11.43	C15H18O4	262.1184	1,3,4-Oxadiazol-2 (3H)-one
11	21 528		M − H = 514 2,881	−7 27	C26H45NO7S	515 2,917	Taurocholic acid
12	22.129	M + Na = 321.2408		−3.56	C18H34O3	298.2508	Ricinoleic acid
13	22.462	M + H = 303.2312		1.76	C20H30O2	302.2246	Abietic acid
14	22.936		M − H = 293.1462	0.57	C10H22N4O6	-	Ethylenediamine diaceturate
15	23.073	M + H = 429.2410	M − H = 427.2334	0.99	-	-	Rhodomollein III
16		M + H = 229 1209		5 53	C15H16O2	228 115	Myrrhone
17	23 715	M + H = 466 3176	M − H = 464 3094	0 13	C26H43NO6	465 309	Glycocholic acid
18	25.193	M + Na = 223.1671		−1.27	C12H24O2	200.1776	Decyl acetate
19	25.922	M + NH4 = 274.2741		-0.07	C16H32O2	256.2402	Lauric acid isobutyl ester
20	26 303		M + H = 229 1214	3 98	C15H16O2	228 115	Myrrhone
21	26.696		M + Na = 339.2518	−3.74	C18H36O4	316.2604	9,10-Dihydroxystearic acid
22	26.684	M + H = 500.3032	M − H = 498.2992	−0.08	C26H45NO6S	499.2968	Taurodeoxycholic acid
23	26.701	M + NH4 = 426.3198		4.47	C24H40O5	408.2876	Cholic acid
24	27.79	M + H = 450.3223	M − H = 448.3154	0.96	C26H43NO5	449.3141	Glycochenodeoxycholic acid
25	27.91	M + Na = 181.1209		−5.73	C9H18O2	158.1307	1-Octanol
26	29 135	M + Na = 305 2,447		1 79	C18H34O2	282 2,259	Oleic acid
27	29 751	M + H = 303 2,314		−0 32	C20H30O2	302 2,246	Abietic acid
28	30.461	M + H = 363.2529		1.88	C22H34O4	362.2437	Fritillebic acid
29	30.773	M + H = 259.1329		−0.2	C16H18O3	258.1256	Broussonin B
30	31.239	M + H = 213.1271		0.83	C15H16O	212.1201	[2-(2-phenylethyl)phenyl]methanol
31	31.819	M + H = 305.2472		1.15	C20H32O2	302.2246	Eicosanetetraenoic acid
32	34.370	M + H = 231.1365		5.85	C15H18O2	230.1307	Curzerenone
33	34 697	M + H = 229 1225		−0 83	C15H16O2	228 115	Myrrhone
34	35.248	M + H = 471.3471	M − H = 469.3372	−0.08	C30H46O4	470.3396	11-Oxo-β-boswellic acid
35	36.076	M + H = 453.2475	M − H = 455.4603	1.02	C24H36O8	452.241	Diacetoxytetrahydr oxytaxadiene
36	37.981	M + H = 347.2589		−2.06	C22H34O3	346.2513	Anacardic acid B
37	38.908	M + H = 513.3575		1.03	C32H48O5	512.3502	Acetyl-11-keto-β-boswellic acid

### XHP exhibited cytotoxicity of PC3 and LNCaP cells

**FIGURE 5:**
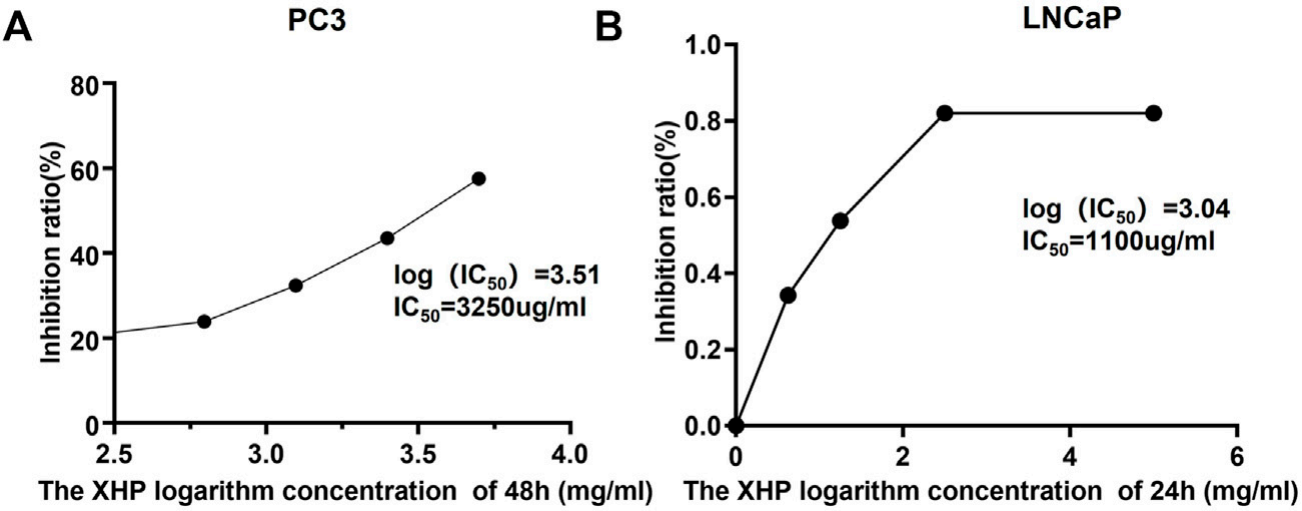
The IC_50_ of XHP to PC3 and LNCaP cells in 48 and 24 h, respectively.(IC_50_, The half maximal inhibitory concentration).

**FIGURE 6:**
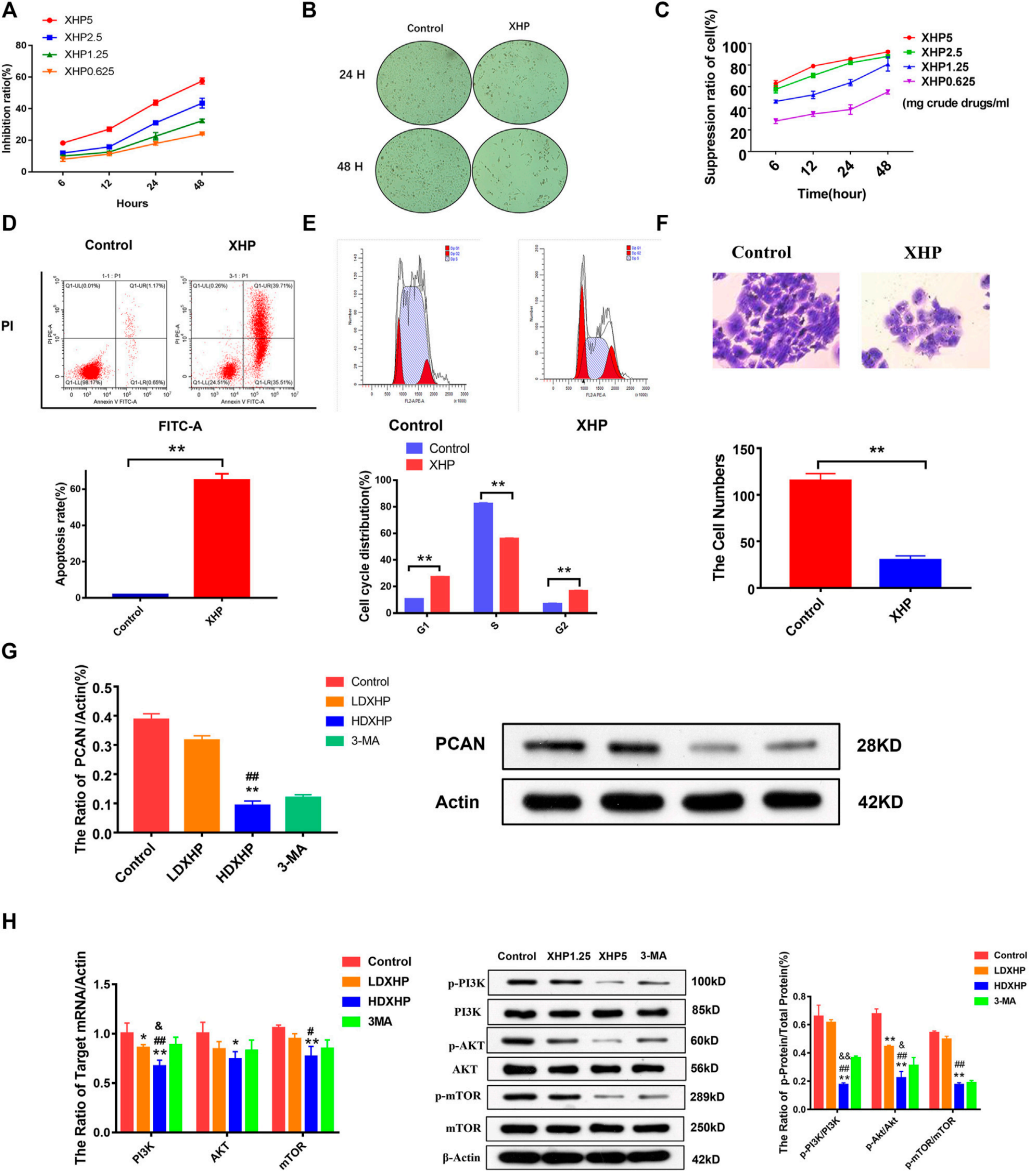
The effect and molecular mechanism of XHP on the proliferation, apoptosis, cell cycle, and invasion of prostate cancer cells. **(A)** The effect of different concentrations of XHP extract on the proliferation of PC3 cells at different times. **(B)** The effects of XHP extract on the proliferation and morphology of PC3 cells. **(C)** The effect of different concentrations of XHP extract on the proliferation of LNCaP cells at different times. **(D)** XHP promoted apoptosis of PC3 cells. **(E)** XHP exhibited cell cycle. **(F)** XHP exhibited invasion against PC3 cells. **(G)** XHP suppressed the protein expression of PCAN in PC3 cells. **(H)**: XHP restrained the protein and mRNA expression of PI3K, Akt, mTOR, p-pI3K, p-Akt, and p-mTOR in PC3 cells [LDXHP: low-dose XHP (1.25 mg/ml); HDXHP: high-dose XHP (5 mg/ml); *compared to the control group, p ≤ 0.05; **compared to the control group, p ≤ 0.01; ^#^compared to the low dose XHP(LDXHP) group (1.25 mg/ml), p ≤ 0.05; ^##^compared to the low dose XHP(LDXHP) group, p ≤ 0.01; ^&^compared to the 3-MA group, p ≤ 0.05; ^&&^compared to the 3-MA group, p ≤ 0.01].

In order to explore the inhibitory effect of XHP on castration-resistant prostate cancer and hormone-sensitive prostate cancer, different concentrations of XHP [5, 2.5, 1.25, and 0.625 mg/ml (crude drug)] were used to treat PC3 cells (castration-resistant prostate cancer) and LNCaP cells (hormone-sensitive prostate cancer) for 6, 12, 24, and 48 h, respectively. CCK8 experiments were used to verify the inhibitory effect of XHP on different prostate cancer cells. As shown in Figures 5A,B, the concentration and time of XHP to reach IC_50_ for different types of prostate cancer cells are different. In this study, the concentration IC50 of XHP on prostate cancer PC3 cells was 3250ug/ml at 48 h. The 24 h IC_50_ concentration of XHP on prostate cancer LNCaP cells was 1100 µg/ml. The results showed that XHP had a well inhibitory effect on hormone-sensitive prostate cancer cells in a short period time, and the enhancement time also had an ideal inhibitory effect on PC3 cells. As shown in Figures 6A-C, the XHP exhibited cytotoxicity against PC3 and LNCaP cells in manners of dose and time-dependence.

### XHP promoted apoptosis, exhibited cell cycle, and invasions against PC3 cells

The Annexin V-APC staining assays, the Canto II flow cytometer, and the *trans*-well invasion assays were performed to analyze the apoptotic, cell cycle, and invasiveness features of PC3 cells treated with XHP for 24 h. As shown in Figure 6D, the apoptosis rate of the XHP group was significantly increased, compared with the control group; the p value (0.004) was calculated by Kruskal-Wallis test (n = 3) (p < 0.01). As shown in Figure 6E, XHP could block cell cycle from G1 phase to S phase, and the difference was statistically significant compared with the control group; all the p values (0.000) of different groups were calculated as by *t*-test (n = 3) (p < 0.01). As shown in Figure 6F, XHP could restrain the invasiveness of PC3 cells and the difference was statistically significant compared with the control group; the p value (0.004) was calculated by Kruskal–Wallis test (n = 3) (p < 0.01).

### XHP restrained the expression of PI3K/Akt/mTOR-related protein and mRNA and PCAN protein in PC3 cells

According to the above cell phenotype experiments, XHP can significantly promote cell apoptosis, block cell cycle, and inhibit cell invasion. In order to further verify the mechanism of XHP to treat the prostate cancer, the molecular mechanism experiments were adopted. The PC3 cells were treated with 1.25 mg/ml and XHP 5 mg/ml of XHP in 24 h, respectively; the blank serum group and 3-MA group were adopted as the control groups. As shown in Figures 6G,H, the HDXHP extracting solution restrained the p-PI3K, p-Akt, p-mTOR, PCAN protein, PI3K, Akt, and mTOR mRNA of PC3 cells significantly, compared to the control, LDXHP, and 3-MA groups (one-way ANOVA test and *t*-test) (p < 0.05). This study suggests that XHP can inhibit the activity of prostate cancer cells and affect the molecular mechanism of intercellular communication.

### XHP inhibited the growth of prostate cancer PC3 cells transplanted tumor *in vivo*

**FIGURE 7:**
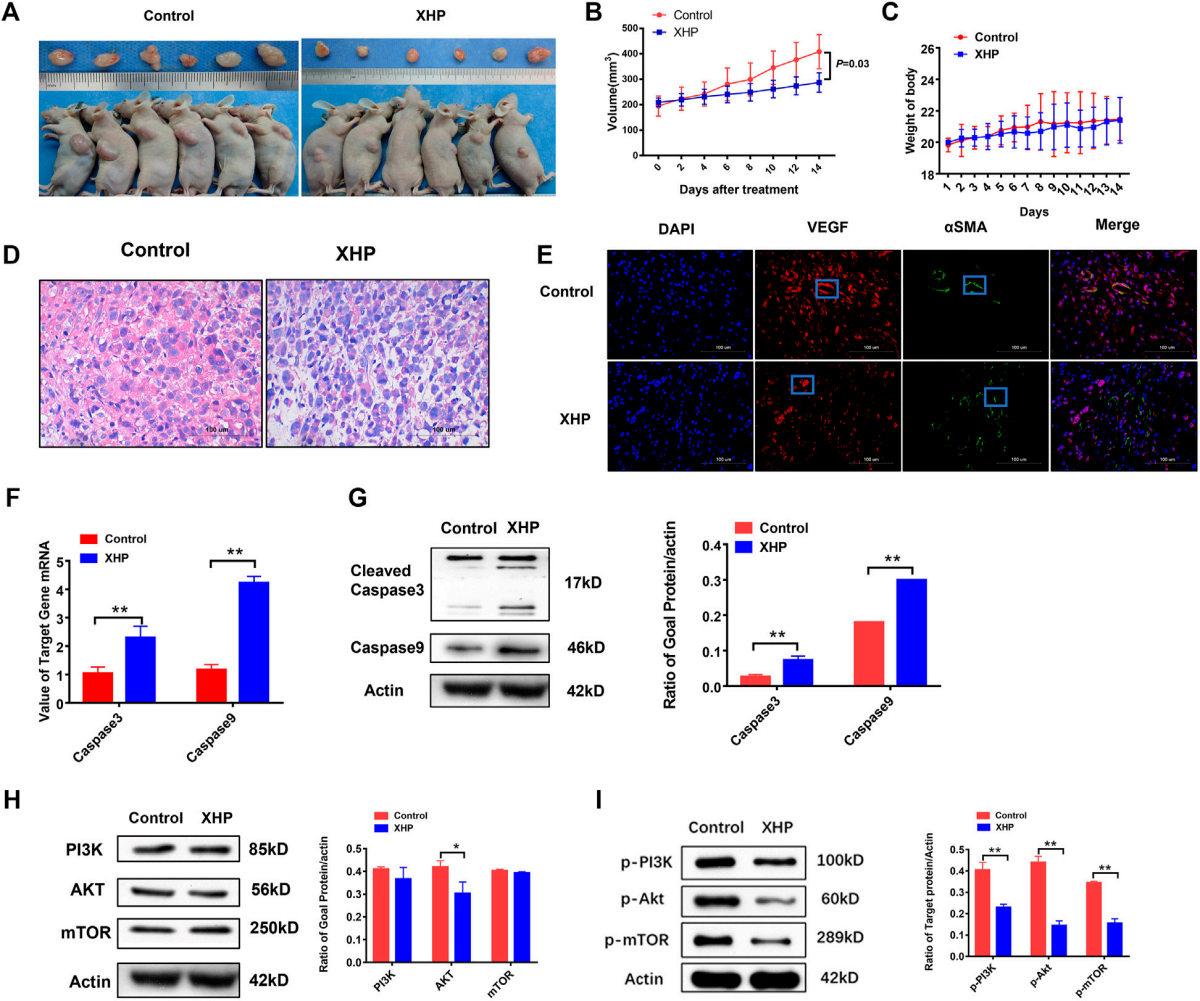
**(A,B)**: The effect of XHP on the volume of prostate cancer PC3 subcutaneous transplantation; **(C)**: The effect of XHP Pill on the weight of prostate cancer PC3 subcutaneous transplantation mice; **(D)**: The effect of XHP on the pathological changes of prostate cancer PC3 subcutaneous transplantation in different groups; **(E)**: Effects of XHP on VEGF and α SMA in different groups of prostate cancer PC3 subcutaneous transplantation. (The red node represents the VEGF signal; The green node represents the αSMA signal). **(F,G)**: Effect of XHP on the protein and mRNA of caspase3 and caspase9 in PC3 subcutaneous transplantation. **(H)**: The effect of XHP on PI3K, Akt, mTOR protein of prostate cancer PC3 subcutaneous transplantation; **(I)**: The effect of XHP on the protein of p-PI3k, p-Akt and p-mTOR in prostate cancer PC3 subcutaneous transplantation. (*Compared to the control group, p ≤ 0.05; **compared to the control group, p ≤ 0.01).

In order to assess whether XHP inhibited the tumor growth *in vivo*, xenograft mouse models were established by subcutaneous transplantation injection of PC3 cells into BALB/C male nude mice. As shown in Figures 7A–C, daily treatment of XHP at the concentration of (78 mg powder/kg/day) for 2 weeks could significantly inhibit the tumor growth in subcutaneous transplantation of prostate cancer PC3 cells (p = 0.03) (*t*-test). There was no significant change in body weight between the control group and XHP group (p > 0.05) (*t*-test). The results showed that XHP had satisfactory inhibitory effect on prostate cancer *in vivo*.

### XHP inhibited the pathological and vascular factors of PC3 cells subcutaneous transplantation

In order to observe the effect of XHP on the pathological tissue of subcutaneous transplantation of prostate cancer, the HE staining microscope was used to evaluate the changes of pathological tissue. The results showed that the tumor cells in the control group had irregular morphology, large and highly stained nuclei, more mitotic images, less cytoplasm, imbalance of nucleo-plasma ratio, unclear cell membrane, less stromal components, and hematopoietic processes between the beam and cable. As shown in Figure 7D, XHP could suppress the staining of tumor nucleus and the distribution of blood vessels in the prostate cancer stroma.

In order to evaluate the effects of XHP on the microenvironment of blood vessels and stroma in the subcutaneous transplantation of prostate cancer, the subcutaneous transplantation tissues were taken for immunofluorescence to observe vascular-related indicators VEGFR and αSMA. As shown in Figure 7E, XHP could inhibit the VEGFR level and increased αSMA level of subcutaneous transplantation of PC3 cells.

### XHP suppressed the protein and mRNA expression of PI3K, Akt, mTOR, p-pI3K, p-Akt, and p-mTOR and increased the protein and mRNA expression of cleave-caspase3 and caspase9 in PC3 subcutaneous transplantation

In order to further verify the mechanism of XHP to treat the prostate cancer, the vivo experiments were adopted. The PC3 subcutaneous transplantation mice were treated with XHP at 78 mg/kg per day for 2 weeks. As shown in Figures 7F,G, XHP could activate the cleave caspase-3 and caspase-9 protein and mRNA of the PC3 prostate cancer subcutaneous transplantation significantly, compared to the control group, p = 0.002. Moreover, as shown in Figures 7H,I, XHP could suppress the Akt (p = 0.024), p-PI3K(p = 0.000), p-Akt (p = 0.000), and p-mTOR (p = 0.000) protein of the PC3 prostate cancer subcutaneous transplantation significantly (p < 0.05) (*t*-test). These results confirm that XHP can promote apoptosis of prostate cancer cells and is related to the PI3K/Akt/mTOR signaling pathway.

## Discussion

Prostate cancer is a serious life-threatening disease of males (Van Den Eeden et al., 2018) and a common male malignancy clinically. It ranks first in male cancer and the incidence rate is second (Miller et al., 2019). Therefore, it is urgent to improve the curative effect of prostate cancer and reduce the mortality of the disease. XHP are a classic anti-cancer prescription and widely used in the treatment of various kinds of tumors (Wang et al., 2021). It comes from *Wai Ke Quan Sheng Ji* in Qing Dynasty. The whole prescription is composed of calculus bovis, musk, frankincense, and myrrh. It is commonly applied in the prevention and treatment of breast cancer, cervical cancer, and other tumors (Wu et al., 2020; Xu X et al., 2020), but its effect on prostate cancer and exact mechanism are unclear.

Network pharmacology is a subject based on system biology to analyze the targets of a variety of drugs, components, and diseases (Hopkins, 2008). In this study, the reported active components of four traditional Chinese medicines in XHP were retrieved by using TCMSP and Batman-TCM database, and 37 main components of XHP were analyzed by ULTRA performance liquid chromatography. The key active components of XHP were quercetin and heptanoic acid, etc., according to the results of ULTRA performance liquid chromatography and the construction of an active component-target interaction network. Studies have shown that quercetin plays an anticancer role by regulating the cell cycle and participating in biological processes such as angiogenesis and PI3K-Akt signaling pathway (Lu X. et al., 2020; Wong et al., 2020). Heptanoic acid plays an anti-prostate cancer role through the caspase pathway (Hamid A. A. et al., 2017). Therefore, the active components of XHP play a synergistic anticancer role through multiple channels.

In order to further construct the active components prostate cancer target interaction network, the potential targets of XHP in the intervention of prostate cancer were predicted by gene ontology (GO) biological function analysis and KEGG pathway enrichment. The results showed that the potential pathways and targets of XHP intervention in prostate cancer include PI3K, Akt, VEGF, caspase3 and caspase9, AR, HIF and so on. Abnormal activation of these targets can promote the biological processes of prostate cancer, such as proliferation, metastasis, inflammation, and angiogenesis, and further aggravate the malignant process of prostate cancer (Yang et al., 2020). Prostate cancer is an androgen-related malignant tumor (Shafi et al., 2013). Androgen receptor (AR) plays an important role in the malignant progression and metastasis of prostate cancer (Abd Wahab et al., 2020). AR target gene transcription, mediated by androgen, can promote the proliferation, differentiation, and metastasis of prostate cancer cells (Fang et al., 2016). Therefore, AR therapy is one of the main means of prostate cancer treatment (Nevedomskaya et al., 2018). However, long-term ADT treatment can abnormally activate the PI3K/Akt pathway (Pisano et al., 2021), thereby enhancing the antiapoptotic ability of tumor. Therefore, the PI3K/Akt pathway is an important potential target of non-AR pathway in the treatment of prostate cancer. In the network pharmacology analysis, according to the KEGG pathway enrichment analysis results, PI3K/Akt related pathway was the pathway with the largest number of KEGG enrichment targets. Therefore, based on PI3K/Akt pathway, this paper will verify the potential mechanism of XHP in the intervention of prostate cancer *in vivo* and *in vitro*. AR and HIF pathways are also predicted to be important targets of XHP in prostate cancer intervention in target prediction, GO function analysis, and gene enrichment analysis. As the above targets also play an important role in the malignant process of prostate cancer, we will verify the above potential targets in the following paper.

Castration-resistant prostate cancer (CRPC) is the focus and difficulty of clinical treatment, with short median survival and poor prognosis (Adashek et al., 2019; Chen et al., 2019). Surgical castration or drug castration is difficult for CRPC (Heidegger et al., 2020). At present, chemotherapy, new second-line endocrine therapy, biotherapy, and other new treatment methods can alleviate the progress of castration-resistant prostate cancer to a certain extent (Giuliano et al., 2019), but there are many disadvantages, such as the small scope of application and difficulty of tolerance for most elderly patients (Italiano et al., 2009; Shevach et al., 2020). Therefore, there is no clear and reliable positive control drug for this experiment. This study will use the model control group and XHP group for comparative study.

As shown in the results of *in vivo* and *in vitro* experiments, XHP inhibited the proliferation of the prostate cancer PC3 and LNCaP cells, promoted the apoptosis, blocked the cell cycle, and restrained the invasion of prostate cancer PC3 cells. What is noteworthy is that the inhibitory effect of XHP on different prostate cancer cell lines was discrepancy. XHP can inhibit hormone-sensitive prostate cancer cell line LNCaP cells well, and its molecular mechanism is worth further exploring. Invasion and metastasis are the significant phenotypes of malignant tumors (Valcarcel-Jimenezet al., 2019). Studies have shown that the high expression of PCAN is associated with the high proliferation and invasiveness of tumor cells (Cheng et al., 2018; Yu et al., 2020). It was confirmed by the invasion experiments that XHP could reduce the invasiveness of prostate cancer PC3 cells and were associated with the decrease of PCAN protein level. The results showed that XHP could downregulate the PI3K/Akt pathway-related proteins in prostate cancer cells and tumor tissues in a dose-dependent manner. PI3K/Akt is an oncogene-related product, which is abnormally activated in a variety of malignant tumors and regulates the proliferation of tumor cells (Hoxhaj and Manning BD, 2020). It plays an important role in promoting the occurrence and development of tumors by inhibiting cell apoptosis, blocking the cell cycle, and recruiting vascular endothelial growth factor (Araki and Miyoshi Y, 2018; Noorolyai et al., 2019; Tan, 2020). Mammalian target of rapamycin (mTOR) is one of the downstream targets of the PI3K/Akt signaling pathway. It is an intermediate regulatory site in biosynthesis and decomposition. It plays a key role in regulating energy metabolism, cell cycle progression, cell proliferation, apoptosis, and autophagy (Wang and Zhang, 2019; Xu Z et al., 2020), and is involved in the occurrence, invasion, and distant metastasis of prostate cancer (Giguère, 2020; Makwana et al., 2021). VEGF and caspase-related proteins are also important downstream targets of the PI3K/Akt pathway, which are the main regulatory points of tumor angiogenesis and apoptosis (Massihnia et al., 2016). It was confirmed that XHP could inhibit PI3K, Akt, mTOR, p-PI3K, p-Akt, and p-mTOR *in vivo* and *in vitro*. Immunofluorescence assay showed that XHP could downregulate the level of VEGFR in prostate cancer tissue. VEGF is an important regulator of angiogenesis (Melincovici et al., 2018). Inhibition of VEGF angiogenesis plays an important role in delaying tumor proliferation and metastasis and improving the tumor microenvironment (Sui et al., 2017). Through the pathological results of this experiment, it was confirmed that the tumor cells in the model control group grew well with irregular shape, unclear cell membrane, less interstitial components, and blood processes between the trabeculae. After XHP intervention, the nuclear staining of the tumor in drug group was weaker than that in the control group, the nuclear was smaller, and the distribution of blood vessels in the stroma was reduced. Therefore, XHP can regulate the PI3K/Akt-related pathway and downstream VEGF protein; promote cell apoptosis; block the cell cycle; inhibit angiogenesis; and then inhibit the proliferation, differentiation, and metastasis of prostate cancer cells.

Apoptosis escape is one of the 10 characteristics of malignant tumors, and it enhances the ability of tumor proliferation and invasion (Zhang et al., 2020). Caspase-3, caspase-9 protein, and other apoptosis pathways are the main executors of PI3K/Akt and other apoptosis pathways, which play a central role in the process of apoptosis (Li et al., 2019). The activation of the target protein induces apoptosis from the mitochondrial pathway and then inhibits the proliferation of tumor cells (Yang et al., 2019; Naz et al., 2020). In this paper, we first predicted that one of the important targets of XHP in the treatment of prostate cancer is caspase protein. Further experimental verification showed that the expression of caspase-3 and caspase-9 protein in prostate cancer tissue was low. After XHP intervention, the above two kinds of apoptotic proteins were upregulated. The results showed that XHP could upregulate the apoptotic protein and promote the apoptosis of prostate cancer cells.

In conclusion, this study predicted the active components of XHP and the related targets of prostate cancer through network pharmacology, and further verified the effect of XHP on inhibiting prostate cancer cell proliferation, promoting cell apoptosis, blocking the cell cycle, and restraining the invasion through *in vitro* and *in vivo* experiments. XHP increased the activity of caspase-3 and caspase-9 apoptotic protein and mRNA levels and suppressed the PCAN proteins. Furthermore, XHP inhibited the protein and mRNA levels of the PI3K-Akt pathway in a concentration-dependent manner. The confirmation of this experimental study will provide a complementary treatment for prostate cancer in addition to potential therapy, anti-AR therapy, chemotherapy, and other conventional treatment methods, and provide some research ideas and basis for the mechanism of the drug.

## Data Availability

The data sets presented in this study can be found in online repositories. The names of the repository/repositories and accession number(s) can be found in the article/Supplementary Material.
